# The Infrared Radiation Temperature Characteristic of Acupoints of Mammary Gland Hyperplasia Patients

**DOI:** 10.1155/2013/567987

**Published:** 2013-11-14

**Authors:** Juanjuan Zheng, Yi Zhao, Yafang Wang, Shengfang Hu, Ping Lu, Xueyong Shen

**Affiliations:** ^1^Acupuncture and Tuina College, Shanghai University of Traditional Chinese Medicine, 1200 Cailun Road, Shanghai 201203, China; ^2^Longhua Hospital, Shanghai University of Traditional Chinese Medicine, Shanghai 200030, China; ^3^Shanghai Research Center of Acupuncture & Meridian, Shanghai 201203, China

## Abstract

*Objective*. To ascertain pathological information on hyperplasia of mammary glands (HMG) of patients via the infrared radiation temperature of acupoints. *Method*. Patients with HMG and healthy controls were tested using an infrared thermal imager. *Results*. In controls, no significant difference in temperature was observed between points with the same name (*P* > 0.05). The temperature of all tested points was found to be higher in the group with HMG than in that of the healthy controls, except for the left and right Zusanli (ST36). The temperature of the right Rugen (ST18), Guanyuan (CV4), Qihai (CV6), and Hegu (LI4) reached a statistically significant heightened level (*P* = 0.046~*P* < 0.001). The temperature of the Zusanli (ST36) and Hegu (LI4) present on the right side was significantly higher than that of the left (*P* = 0.001 and *P* = 0.004, resp.), while the temperature of the left Youmen (KI21) was significantly higher than that of the right (*P* = 0.008). *Conclusion*. The temperature of the bilateral acupoints in healthy controls was symmetrical, and the raised temperatures observed of the Rugen (ST18), Guanyuan (CV4), Qihai (CV6), and Hegu (LI4) acupoints of HMG patients and the imbalance of the temperature of the bilateral acupoints Zusanli (ST36), Youmen (KI21), and Hegu (LI4) carried special pathological information about HMG disease.

## 1. Introduction 

Acupuncture points are sites where qi and the blood of zang-fu organs and meridians are transported. The changes in qi and blood can be reflected in these acupoints. Thus, acupuncture points not only can receive stimulation by acupuncture and moxibustion but also may reflect disorders in patients. Therefore, when there are disorders in zang-fu organs, there could be tenderness, pain, soreness, numbness, or thermosensitivity in the related acupoints. The changes can also be reflected as changes in the biophysical character of the acupoints, such as changes in the volt-ampere characteristics of the points [1, 2] or an infrared radiation intensity change [3]. The human body is a natural source of infrared radiation; it continuously emits infrared radiation into the surrounding environment. When people suffer from ailments or any physiological changes, their systemic or local thermal equilibrium is affected or destroyed. As a result of this, the temperature of their tissues is observed to increase or decrease. Thus, the infrared radiation temperature of acupoints can also carry specific pathological information of diseases related to them. Hyperplasia of the mammary gland (HMG), also known as breast dysplasia, is classified under the “Rupi” category in TCM, which accounts for 75% of all breast disease; ~40% of childbearing women have this disease, which makes it the most common breast disease [4]. With increasing environmental pollution, social pressure, and life-pace acceleration, the incidence of HMG is rising, and the age onset has decreased. Studies have shown that malignant transformation of normal breast tissue undergoes a multistage and gradual process as follows: hyperplasia → dysplasia → carcinoma in situ → invasive cancer, and is considered reversible before developing into invasive cancer [5]. In our research, we study HMG patients and record the infrared radiation temperature of the chosen acupoints to explore its specific pathological information.

## 2. Patients and Methods 

### 2.1. Patients

This study included 101 female HMG patients from the breast clinic of Longhua Hospital, which is affiliated to the Shanghai University of Traditional Chinese Medicine. All of them volunteered to participate in the program and signed informed consent forms. Out of these patients, 73 had bilateral HMG, 13 had only right-lateralized HMG, and 15 were only left-lateralized. The youngest patient was 21 years old and the oldest was 51; the mean age of the sample was 33.14 ± 8.001 years. Thirty-five healthy controls who participated in this study were all interns and staff members of the hospital working at different posts. Seven were medical care workers and the remaining 28 were from house-keeping, finance, and the catering departments. In the control group, the youngest participant was 18 years old and the oldest was 49. The mean age of the control group was 32.23 ± 10.085 years. There was no significant difference in the age distribution between the two groups (*P* = 0.631). Detection time was from September 2008 to November 2008 and from August 2009 to December 2009.

### 2.2. Inclusion and Exclusion

The diagnostic and syndrome differentiation standards adopted in 2002 at the 8th meeting of the Professional Committee for Breast Diseases of China TCM Surgery Society [6] were used in this trial. Patients who met the inclusion criteria were between 18 and 55 years of age. These patients voluntarily participated and showed good compliance when enrolled. Those patients with the following conditions were excluded: mastitis, a simple benign tumour of the mammary gland, a malignant tumour of the mammary gland, or complicated syndromes that were difficult to differentially diagnose. Patients were also excluded if they were pregnant, lactating, had irregular menstruation, functional uterine bleeding, mental disease, were taking endocrine hormones within the previous 6 months, had gynaecomastia, showed mammary development before menarche, or had serious heart, liver, kidney, or hematopoietic system disease.

The control group consisted of women who had no mammary disease or serious heart, liver, kidney, or cerebral disease, were 18–55 years old, had normal menstruation, voluntarily participated, and showed good compliance when enrolled.

### 2.3. Testing Equipment and Environment

For infrared testing, a 5th generation high-performance uncooled focal plane economic infrared thermograph ThermaCAMTMP30 (FLIR, Sweden) was used. It uses the focal plane array (FPA) principle, an uncooled microbolometer, and has ultra-high thermal sensitivity (0.08°C). The temperature measuring range is 0–500°C; the IFOV, 1.3 mrad; and the wavelength range, 7.5–13 *μ*m. This study was conducted at room temperature of 22 ± 3°C and at an air humidity of 55 ± 10%. There was no obvious air flow, strong noise, or electromagnetic sources around.

### 2.4. Testing Acupoints and Methods

According to the national standard GB12346-90 Acupoints Location released by the State Bureau of Technical Supervision in 1990 [7], we chose 7 relevant acupoints in HMG patients for infrared thermograph: Rugen (ST18), Qimen (LR14), Youmen (KI21), Guanyuan (CV 4), Qihai (CV 6), Zusanli (ST 36), and Hegu (LI4). In addition, bilateral acupoints sharing the same name were used. Therefore, a total of 12 points were tested. 

**Figure 1:**
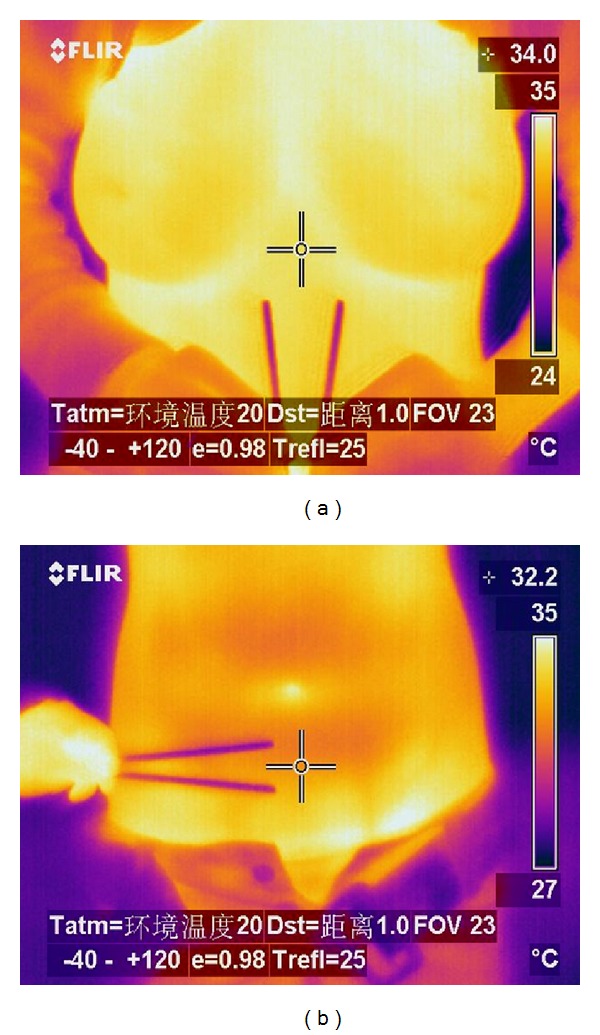
Infrared radiation temperature of acupoints. Bamboo stick in picture (a) points to Youmen (KI21) on both sides. In picture (b), it points to Guanyuan (CV4) and Qihai (CV6). The centre of the cross indicates the focus when the picture was taken. Data written on the top right of the picture is the temperature of the point.

Patients entered the lab, loosened their underwear, and sat down for 20 min to adapt to the environment. During this period, they are asked questions to obtain the relevant required information. After questioning, the patients exposed their testing sites, and the analyser sat 1 meter away from the testing site in a fixed position prepared to film the procedure. The infrared camera was placed right above the testing site to obtain infrared images.  Figure 1 is an infrared image of acupoints on the upper and lower abdomen. 

### 2.5. Statistical Method

The thermaCAM reporter 2000 professional analysis system, matching the infrared thermography, was used to analyse the infrared thermograph picture and obtain the skin temperature at the detected acupoints; Microsoft Excel was used to construct a database and the SPSS 11.5 (SPSS Inc., Chicago) software package was used to analyse the statistical data. We used mean and standard deviation to analyse the measurement data, group *t*-tests for comparison between patients and healthy controls, and paired *t*-tests for comparison between bilateral acupoints with the same name (*α* = 0.05).

### 2.6. Results of the Comparison of Infrared Radiation Temperature between the Healthy Controls and HMG Patients

Except for bilateral Zusanli (ST36), the infrared radiation temperature of 10 out of 12 testing points was higher than that of the healthy controls. Among these 10 testing points, the temperature of the right Rugen (ST18), Guanyuan (CV4), Qihai (CV6), and bilateral Hegu (LI4) was significantly higher than that of the healthy controls (*P* = 0.046~*P* < 0.001) (Table 1). 

### 2.7. The Infrared Radiation Temperature Comparison of Bilateral Acupoints That Share the Same Name in Healthy Controls

Out of 5 testing acupoints in healthy controls, the infrared radiation temperatures of Rugen (ST18) and Qimen (LR14) on the left side were higher than those on the right side, while the temperature of the other 3 acupoints on the right side was higher than those on the left side. However, the difference was not statistically significant (*P* > 0.05) (Table 2). 

### 2.8. The Infrared Radiation Temperature Comparison of Bilateral Acupoints That Share the Same Name in HMG Patients

Out of 5 testing acupoints in patients, the infrared radiation temperature of Rugen (ST18), Qimen (LR14), and Youmen (KI21) on the left side was higher than that on the right side, while the temperature of the other 2 acupoints was higher on the right side than that on the left. The temperature of Youmen (KI21) on the left side was significantly higher than that on the right side (*P* = 0.008), while the temperature of Hegu (LI4) and Zusanli (ST36) on the right side was significantly higher than that on the left side (*P* = 0.001, *P* = 0.004) (Table 3). 

## 3. Discussion 

It's recorded in the *Zhen Jiu Jia Yi Jing* that: “Therapy of breast pain and swelling of the chest lies in the Rugen point.” *Pu Ji Fang* mentioned that “Therapy of breast pain lies in Rugen point.” Nowadays, many textbooks [8–11] take Rugen (ST18) as an important acupoint to treat Rupi, and clinical reports [12, 13] have proven that Rugen (ST18) is particularly effective in HMG treatment. Qimen (LR14) is the front-mu point of the liver meridian. Our previous research on the infrared radiation spectrum found that, to some extent, Qimen (LR14) reflects the pathological nature of the liver qi stagnation of HMG patients [14]. Youmen (KI21) is the crossing point of the kidney meridian and the Chong meridian that is in close proximity to the breast. Guanyuan (CV4) and Qihai (CV6) are important acupoints in the Ren meridian. Guanyuan (CV4), Qihai (CV6), and Youmen (KI21) can reflect the disturbances of HMG patients [15] in the Chong and Ren meridians. Zusanli (ST36) is an important acupoint in the stomach meridian of Foot Yangming. This meridian travels down the inside of the breast. There have been reports showing that Zusanli (ST36) is an important acupoint in HMG therapy [16]. Therefore, in this research, we selected Rugen (ST18), Qimen (LR14), Youmen (KI21), Guanyuan (CV4), Qihai (CV6), Zusanli (ST36), and Hegu (LI4) as testing points. Except for bilateral Zusanli (ST36), the detected infrared radiation temperature of the other 10 points was higher than that of healthy controls. The temperature of the right Rugen (ST18), Guanyuan (CV4), Qihai (CV6), and bilateral Hegu (LI4) reached statistical significant levels (*P* < 0.05). Researchers [17, 18] reported that thermography of hyperplasia mammary gland is manifested as a cluster and sheet-shaped high-temperature hot zone. Its peripheral area is shown as a low heat area, and it has clear but irregular boundaries and an ectatic vascular shadow. Its distribution is continuous and irregular. The raised temperature of the areola is the most obvious, making it the high-temperature area. This is basically consistent with the temperature trend of acupoints on the trunk as reflected in this test result. It points out that the overall pathological condition of HMG patients exceeds that of controls. Infrared radiation temperature of the right Rugen (ST18) was significantly higher than that of healthy controls, which reflected the raised temperature phenomenon of the local breast tissue. The temperature of Guanyuan (CV4) and Qihai (CV6) on the Ren meridian was significantly higher than that of healthy controls, which may be a reflection of the disorders of the Chong and Ren meridians. Hegu (LI4) is the source point of the large intestine meridian, and the temperature of this point is significantly higher than that of healthy controls. We believe that there are two possibilities: first, the findings of the clinical observations suggest that many HMG patients often have gastrointestinal disorder symptoms such as constipation, which could be reflected on Hegu (LI4) of the large intestine meridian; second, pain is a primary clinical symptom of HMG patients, and the temperature-rising phenomenon is likely to be an important reaction to breast pain.

There have been ancient records for Hegu (LI4) on the treatment of painful diseases. *Xi Hong Fu *says: “Unbearable pain of the hands and shoulders should be treated with Hegu and Taichong.” *Sheng Yu Ge* says: “Soreness and pain of the hands, which makes it hard to hold things, should be treated with Hegu, Quchi, and Jianyu.” There are also reports on Hegu (LI4) for pain treatment in a modern clinic [19–22]. Researchers [23] proved that electric acupuncture on Hegu (LI4) can elicit compound action, respectively, on trigeminal nerve branches, and the pain threshold of the head, chest, and abdominal skin rapidly becomes higher. This phenomenon disappears when we completely block the points. So, it is suggested that Hegu (LI4) is an important acupoint for treating pain and an important reflection point of breast pain. According to the human body symmetry, in order to measure the temperature difference of human bilaterally corresponding parts with thermal images, determination of the occurrence of disease is a common diagnostic method of thermal imaging. Feldman filmed thermal images of the cervical vertebrae and upper limbs of healthy people in 1994 [24]. He believed that normal temperature differences of bilateral sides of the upper body would be no more than 0.62°C; Uematsu et al. [25] thought that the temperature difference should be between 0.2 and 0.5°C and the maximum difference is 1°C [26]. Yan's study showed that the surface temperature of a normal human body and that of a rabbit are basically symmetrical, while the spleen-deficiency diarrheogenic rabbit model showed significant asymmetry in temperature change of the left and right side of the body [27, 28]. He present a study that showed that there was no statistically significant difference in the infrared radiation temperature of bilateral points sharing the same name and that of healthy controls (*P* > 0.05). This was consistent with the symmetry law of bilateral, normal, skin temperature, while the infrared radiation temperature difference of bilateral Youmen (KI21), Zusanli (ST36), and Hegu (LI4) of HMG patients was statistically significant (*P* < 0.05). This showed the temperature imbalance phenomenon of bilateral acupoints of HMG patients and indicated the pathological nature of liver qi stagnation, phlegm and blood stasis, and Chong and Ren meridian disturbance of HMG patients. 

## 4. Conclusion

The infrared radiation temperature of the bilateral acupoints in healthy controls was found to be symmetrical, and the raised temperatures observed of the Rugen (ST18), Guanyuan (CV4), Qihai (CV6), and Hegu (LI4) acupoints of mammary gland hyperplasia patients and the imbalance of the infrared radiation temperature of the bilateral acupoints Zusanli (ST36), Youmen (KI21), and Hegu (LI4) carried special pathological information about mammary gland hyperplasia disease. These results can be used as an auxiliary diagnosis for mammary gland hyperplasia. This experiment provides new research ideas and methods for TCM diagnosis using acupoints, the research of acupoint specificity, acupoint selection at the clinic, and the objective research on the evaluation of curative effectiveness.

## Figures and Tables

**Table 1:** Infrared radiation temperature comparison of the tested points of patients and healthy controls ($\overset{-}{x}\pm s$, °C).

Acupoints	Patients	Healthy controls	*t *	*P *

Left Rugen (ST18)	33.888 ± 1.0(*n* = 101)	33.503 ± 1.3(*n* = 35)	1.559	0.126
Right Rugen (ST18)	33.878 ± 0.94(*n* = 101)	33.340 ± 1.5(*n* = 35)	2.053	**0.046 **
Left Qimen (LR14)	33.943 ± 0.9(*n* = 101)	33.811 ± 1.2(*n* = 35)	0.662	0.509
Right Qimen (LR14)	33.871 ± 0.9(*n* = 101)	33.629 ± 1.2(*n* = 35)	1.266	0.208
Left Youmen (KI21)	33.677 ± 1.1(*n* = 100)	33.246 ± 1.5(*n* = 35)	1.615	0.113
Right Youmen (KI21)	33.600 ± 1.0(*n* = 100)	33.286 ± 1.3(*n* = 35)	1.422	0.157
Guanyuan (CV4)	33.756 ± 1.1(*n* = 101)	32.746 ± 1.5(*n* = 35)	3.717	**0.001 **
Qihai (CV6)	33.428 ± 1.1(*n* = 101)	32.543 ± 1.3(*n* = 35)	3.907	**0.000 **
Left Zusanli (ST36)	32.584 ± 0.9(*n* = 101)	32.677 ± 1.0(*n* = 35)	−0.499	0.618
Right Zusanli (ST36)	32.696 ± 1.0(*n* = 101)	32.760 ± 1.0(*n* = 35)	−0.334	0.739
Left Hegu (LI4)	32.781 ± 1.2(*n* = 97)	32.160 ± 1.3(*n* = 35)	2.539	**0.012 **
Right Hegu (LI4)	32.904 ± 1.2(*n* = 97)	32.263 ± 1.3(*n* = 35)	2.595	**0.011 **

**Table 2:** Infrared radiation temperature comparison of bilateral acupoints with the same name of healthy controls ($\overset{-}{x}\pm s$, °C).

Acupoints	Left side	Right side	*t *	*P *
Rugen (ST18) (*n* = 35)	33.503 ± 1.3	33.340 ± 1.5	1.568	0.126
Qimen (LR14) (*n* = 35)	33.811 ± 1.2	33.629 ± 1.2	1.648	0.109
Youmen (KI21) (*n* = 35)	33.246 ± 1.5	33.286 ± 1.3	−0.824	0.416
Zusanli (ST36) (*n* = 35)	32.677 ± 1.0	32.760 ± 1.0	−1.370	0.180
Hegu (LI4) (*n* = 35)	32.160 ± 1.3	32.263 ± 1.3	−1.135	0.264

**Table 3:** Infrared radiation temperature comparison of bilateral acupoints with the same name of patients ($\overset{-}{x}\pm s$, °C).

Acupoints	Left side	Right side	*t *	*P *
Rugen (ST18) (*n* = 101)	33.888 ± 1.0	33.878 ± 1.9	0.217	0.829
Qimen (LR14) (*n* = 101)	33.943 ± 1.0	33.871 ± 0.9	1.592	0.114
Youmen (KI21) (*n* = 100)	33.677 ± 1.1	33.600 ± 1.0	2.709	**0.008 **
Zusanli (ST36) (*n* = 101)	32.584 ± 1.0	32.696 ± 1.0	−3.513	**0.001 **
Hegu (LI4) (*n* = 97)	32.781 ± 1.2	32.904 ± 1.2	−2.928	**0.004 **
